# Genome-Wide Identification and Comprehensive Analysis of the GS Gene Family in *Hordeum vulgare* Under Low Nitrogen Stress

**DOI:** 10.3390/biology14121789

**Published:** 2025-12-15

**Authors:** Yaping Pei, Juncheng Wang, Lirong Yao, Erjing Si, Ke Yang, Baochun Li, Yaxiong Meng, Xiaole Ma, Hong Zhang, Xunwu Shang, Huajun Wang

**Affiliations:** 1 State Key Laboratory of Aridland Crop Science, Gansu Key Lab of Crop Improvement and Germplasm Enhancement, Lanzhou 730070, China; 2 Department of Crop Genetics and Breeding, College of Agronomy, Gansu Agricultural University, Lanzhou 730070, China; 3 Department of Botany, College of Life Sciences and Technology, Gansu Agricultural University, Lanzhou 730070, China

**Keywords:** *Hordeum vulgare*, glutamine synthetase, low nitrogen stress, genome-wide analysis, nitrogen use efficiency

## Abstract

Glutamine synthetase (GS; EC 6.3.1.2) is a key enzyme for primary assimilation and re-assimilation of ammonium in higher plants. Although several GS gene families have been reported for several cereal crops, systematic studies for barley (*Hordeum vulgare*) under different nitrogen treatment conditions are still lacking. In this study, we combined genome-wide bioinformatics mining with transcriptome analysis to characterize the *HvGS* gene family in two different genotypes of barley (nitrogen-efficient W26 and nitrogen-sensitive W20) and their responses to low nitrogen stress. Four *HvGS* genes were retrieved from the barley genome and named *HvGS*1–*HvGS*4. These genes were comprehensively analyzed in terms of chromosomal distribution, physicochemical properties, subcellular localization, intron-exon structure, conserved motifs, promoter cis-acting elements, evolutionary relationships, and predicted protein–protein interactions. Leaves and roots were sampled and subjected to RNA-seq analysis at 3, 18, and 21 days of low-nitrogen stress, which revealed significant expression differences among genotypes and tissues. In W26, low nitrogen (0.4 mmol·L^−1^) induced synergistic expression of *HvGS*1 and *HvGS*4 and suppressed expression of plastidic *HvGS*2, whereas W20 up-regulated the expression of *HvGS*1 and *HvGS*3 mainly in the root system. Combined GO/KEGG enrichment analysis and metabolomic characterization of the differentially expressed genes highlighted nitrogen metabolism, glutathione turnover, and amino acid biosynthesis as key hubs in the tolerant genotypes. Our results provide a genome-wide analysis of the barley GS family and highlight *HvGS*1 and *HvGS*4 as candidate genes for functional validation toward improved nitrogen use efficiency.

## Simple Summary

Nitrogen fertilizer is widely used to increase crop yields, but much of it is wasted, causing pollution and raising production costs. Barley is an important cereal crop, and improving how barley plants use nitrogen can help farmers maintain yields with less fertilizer. In this study we searched the barley genome for all genes that encode glutamine synthetase, a key enzyme that converts inorganic nitrogen into amino acids that plants can use for growth. We then compared how these genes behaved in two barley varieties that differ in their ability to cope with low nitrogen supply, growing plants in nutrient solutions with normal and reduced nitrogen and then restoring nitrogen. We found four glutamine synthetase genes and showed that they are switched on in different tissues and at different stages of nitrogen stress and recovery. The variety with efficient nitrogen uses coordinated changes in gene activity and metabolism to maintain leaf nitrogen assimilation under low nitrogen supply. These findings provide useful targets for breeding barley varieties that need less fertilizer and cause less environmental impact.

## 1. Introduction

Nitrogen fertilizer and nitrogen fertilizer play key roles in crop growth and development and crop yield, respectively [1]. However, excessive application of nitrogen fertilizer can cause serious environmental pollution [1,2]. Therefore, high nitrogen utilization efficiency (NUE) is essential for increasing crop yield and reducing environmental pollution [3,4]. In plants, NUE is a complex trait associated with nitrogen accumulation and metabolism, including uptake, transport, assimilation, and remobilization or reuse [5,6,7]. Plants usually take up N in inorganic (nitrate and ammonium) or organic (amino acids and urea) forms [5,8,9]. Rice is a typical ammonium-loving crop, while barley takes up nitrogen primarily in the form of nitrate [10,11]. Enzymes involved in nitrogen assimilation include nitrate reductase (NR), nitrite reductase (niR), glutamine synthetase (GS), glutamate synthetase (GOGAT), glutamate dehydrogenase (GDH), glutamate decarboxylase (GAD), and asparagine synthetase (ASNS) [12,13,14]. Two biosynthetic pathways have been proposed in plants: glutamate (Glu) and ornithine (Orn) pathways [15,16]. In the Glu pathway, Pro biosynthesis begins with the phosphorylation of Glu to form γ-glutamyl phosphate, which is converted by the bifunctional enzyme Δ1-pyrroline-5-carboxylate synthetase (P5CS; EC 2.7.2.11/1.2.1.41) to glutamate-5-semialdehyde (GSA), which is spontaneously cyclized to pyrroline-5-carboxylate (P5C) [15]. Finally, by Δ1-pyrroline-5-carboxylate reductase (P5CR; EC 1.5.1.2) [15]. In this pathway, Pro biosynthesis occurs in the cytoplasm and chloroplasts, and glutamate is mainly derived from the glutamine synthetase-glutamine oxoglutarate aminotransferase (GS-GOGAT) cycle [15]. In the Orn pathway, Orn passes through ornithine δ-transferase (δ-OAT; EC 2.6.1.13) and is subsequently converted to Pro via P5C. It has been shown that Pro biosynthesis in Arabidopsis occurs exclusively via the Glu pathway [16]. Therefore, the Oen pathway remains controversial [16]. Under osmotic stress, the Glu pathway is enhanced by the enhancement of the Under osmotic stress, Pro biosynthesis via the Glu pathway, which enhances the GS-GOGAT cycle, is the main pathway [15,16].

Glutamine synthetase (GS; EC 6.3.1.2, L-glutamate: ammonia ligase ADP formation) is the key enzyme responsible for primary nitrogen (N) assimilation in higher plants [17,18]. Glutamine synthetase catalyzes the ATP-dependent addition of ammonium (NH_4_^+^) to the γ-carboxyl group of glutamate to produce glutamine and participates in the GS-GOGAT cycle, the cornerstone of nitrogen metabolism [17]. The sources of ammonium assimilated by GS include fixation of atmospheric nitrogen, direct uptake of nitrate or ammonia from the soil, photorespiration, phenylalanine deamination catalyzed by phenylalanine-ammonia cleavage enzymes and ammonium release from the plant during storage through protein mobilization and ammonium release from plant senescence [12,17]. Thus, in the context of nitrogen assimilation, GS is considered a candidate gene for transgenic approaches to improve nitrogen use efficiency (NUE) [18,19,20]. GS also responds to a variety of abiotic stresses, including salt, cold, and drought, which adversely affect crop production [18,19]. Oligomeric isozymes consisting of GS polypeptides encoded by multiple nuclear genes are located in the cytoplasm or chloroplasts and are expressed in non-photosynthetic and photosynthetic tissues of higher plants [17,18,19]. Researchers reported that the decametric structure of plant GS holoenzyme consists of two face-to-face cyclic pentameric subunits [21]. In vascular plants, there are two major isoforms of GS, classified as cytoplasmic GS (GS1) and chloroplast GS (GS2) based on their size and subcellular localization [17,18,22]. Genome analysis of several angiosperm species has shown that GS1 genes belong to a small multigene family, whereas GS2 is encoded by one or two genes [22,23,24]. Cytoplasmic GS1 isoforms take up ammonium from the soil, and ammonia is remobilized and released through protein degradation in senescent leaves, whereas the larger chloroplast-localized GS2 isoforms are responsible for the reassimilation of ammonium released during photorespiration and the reduction in nitrate in the plastids. Different expression patterns of these genes regulate glutamine production spatially and temporally [25]. For example, in rice (*Oryza sativa*), there are three genes encoding cytoplasmic GS1 (OsGS1.1, OsGS1.2, and OsGS1.3) and one encoding plastidic GS2 (OsGS2). osGS1.1 is present globally but is expressed more in the aboveground part of the plant, whereas OsGS1.2 is expressed predominantly in roots. osGS1.3 is a gene encoding cytoplasmic GS1, but is not expressed in the aboveground part. It is almost undetectable except in spikelets, while OsGS2 is abundant in leaves [24].

Studies have shown that GS isozymes have different functions in nitrogen metabolism in wheat. TaGS1 (GS1.1) and TaGSr (GS1.2) are mainly involved in the reutilization of nitrogen in senescent leaves [26]. The GS gene family has been investigated in some plants, including *Arabidopsis thaliana* [25], maize (*Zea mays*) [20] and poplar (*Populus trichocarpa*) [27]. However, the GS gene family has not been systematically reported in barley. Barley is one of the major cereal crops in the world, and high nitrogen utilization is important to increase yield with barley [11]. The aim of this paper was to conduct a comprehensive study on the molecular characterization, phylogenetic relationships and expression profiles of the GS gene family in barley. In addition, we selected a nitrogen-efficient genotyped barley variety (W26) and a nitrogen-sensitive genotyped barley variety (W20), and analyzed the role of *HvGS* on low-nitrogen stress with the help of transcriptome by applying low-nitrogen stress to the two barley varieties.

## 2. Materials and Methods

### 2.1. Plant Materials, Hydroponic Treatments and Sampling

Seeds of the nitrogen-efficient variety W26 and nitrogen-sensitive variety W20 were selected, surface-sterilized, and germinated on moistened filter paper for 48 h at 22 °C under dark conditions. Seedlings with uniform growth were selected and transferred to 1/2 concentration of Hoagland nutrient solution (pH 6.0) (Hopebio, Qingdao, China) and acclimatized for 5 days under controlled growth chamber conditions (22 °C/18 °C Day/night, 16 h photoperiod, 200 μmol·m^−2^·s^−1^ photosynthetic photon flux density). This was followed by hydroponic experiments (Smart Plus E, Heal Force, Shanghai, China) with two nitrogen treatments: normal nitrogen (NN, 2 mmol·L^−1^ total nitrogen as 1.8 mmol·L^−1^ NO_3_^−^ and 0.2 mmol·L^−1^ NH_4_^+^ form) and low nitrogen (LN, 0.4 mmol·L^−1^ total nitrogen in the same NO_3_^−^/NH_4_^+^ ratio). The nutrient solution was changed every 48 h and continuously aerated. After 18 days of low nitrogen treatment, the nitrogen concentration was restored to half of the original concentration to simulate the recovery process for another 3 days (total experimental duration 21 days). Leaf and root tissues were collected from three biological replicates of each genotype × treatment combination on day 3 (early response), day 18 (prolonged stress), and day 21 (recovery) after treatment, respectively. Samples were rapidly frozen in liquid nitrogen and stored at −80 °C for RNA extraction.

### 2.2. Genomic Resources and Gene Identification

Were obtained from the EnsemblPlants and NCBI databases for barley (Morex V3), wheat (*Triticum aestivum* IWGSC RefSeq v2.1), rice (*Oryza sativa* L. japonica Nipponbare), maize (*Zea mays* B73 RefGen_v5), Arabidopsis (*Arabidopsis thaliana* TAIR10), and representative microbial GS proteins for chromosome sequence, gene annotation, and predicted proteome. Known GS proteins were used as query sequences for comparison in the barley proteome by BLASTP (v2.13.0) search (E-value < 1 × 10^−5^, identity > 50%). Conserved structural domain searches were performed through the NCBI CDD and Pfam databases to confirm candidate sequences, and only entries containing both Gln-synt_N (PF00120) and Gln-synt_C (PF03951) structural domains were retained. Redundant isoforms were removed based on the criterion of >98% sequence identity.

### 2.3. Physicochemical Properties, Subcellular Localization and Chromosomal Localization

Protein length, molecular weight, theoretical isoelectric point, instability index, aliphatic index and hydrophilicity averages were calculated using ExPASy ProtParam (https://web.expasy.org/protparam/, accessed on 8 December 2025). Subcellular localization was predicted by TargetP 2.0, WoLF PSORT (https://wolfpsort.hgc.jp/, accessed on 8 December 2025) and CELLO v2.5. Genomic coordinates were extracted from barley GFF3 annotations and visualized on barley chromosomes using TBtools (v2.309).

### 2.4. Gene Structure, Conserved Motifs and Promoter Analysis

Exon-intron structure was displayed using Gene Structure Display Server 2.0. Conserved amino acid motifs were identified using MEME v5.5.4 with the following settings: maximum number of motifs = 10, motif width = 6–50 residues, distribution = zero or one occurrence per sequence. The promoter region (2 kb upstream of the ATG) was scanned for cis-acting elements using PlantCARE (http://bioinformatics.psb.ugent.be/webtools/plantcare/html/, accessed on 8 December 2025). Identified elements were categorized into hormone response, stress response, and growth/development categories.

### 2.5. Phylogenetic Reconstruction and Covariance Analysis

Multiple sequence comparisons of GS proteins from barley and reference species were generated using MEGA’s (v11.0.13) NJ algorithm. Based on paired BLASTP comparisons, covariance and collinearity analyses were performed using MCScanX (v1.0); covariance blocks were visualized using TBtools.

### 2.6. RNA Extraction and qRT-PCR Fluorescence Quantification, Read Segment Alignment, Expression Quantification and Differential Analysis

Total RNA was extracted from the samples using TRIzol reagent (Invitrogen, Carlsbad, CA, USA), taking advantage of FastQuant First Strand cDNA Synthesis Kit (Tiainen, Beijing, China) to synthesize cDNA. These reactions were carried out under the following conditions: 37 °C for 15 min, 85 °C for 5 s, and finally ending at 4 °C. LightCycler 480 Real-Time PCR System (Roche Applied Science, Penzberg, Germany) and SYBR Green Premix Pro Taq HS Premix kit were used for qRT-PCR. The reaction system was 2 × SYBR Green Pro Taq HS Premix 10 μL, primer F 0.4 μL, primer R 0.4 μL, cDNA 2 μL, ddH_2_O 7.2 μL. The primers used in qRT-PCR were designed with Primer 5.0. The qRT-PCR data were analyzed using the 2^−ΔΔCt^ calculation method.

Clean reads were aligned to the Morex V3 reference genome using HISAT2 v2.2.1. Transcript abundance was quantified in units per kilobase exon per million mapped reads (FPKM) using StringTie v2.2.1. Differential expression between treatments was assessed using DESeq2 v1.38 (|log_2_ fold change| ≥ 1, corrected *p* ≤ 0.05). Gene ontology and KEGG pathway enrichment analysis were performed using clusterProfiler v4.6 based on barley-specific annotations.

### 2.7. Statistical Analysis

A minimum of three biological replicates was used for all experiments. Statistical analysis was performed using two-way ANOVA to assess the effects of genotype (W26 vs. W20), treatment (control, low nitrogen, recovery), and their interaction on gene expression. Prior to ANOVA, assumptions of normality (Shapiro–Wilk test) and homogeneity of variances (Levene’s test) were verified. When assumptions were met, two-way ANOVA was performed. When the interaction term was significant (*p* < 0.05), simple effects analysis was conducted. Post hoc multiple comparisons were performed using Tukey’s Honestly Significant Difference (HSD) test. Statistical significance was set at *p* < 0.05. For qRT-PCR data, statistical significance is indicated in the figures using asterisks (* *p* < 0.05, ** *p* < 0.01, *** *p* < 0.001) or “ns” (not significant). All statistical analyses were performed using SPSS version 26.0, R version 4.2.0, OriginPro 2022, and GraphPad Prism 9.

## 3. Results

### 3.1. Genome-Wide Identification, Physicochemical Characterization and Chromosomal Localization of GS Genes in Barley

**Figure 1:**
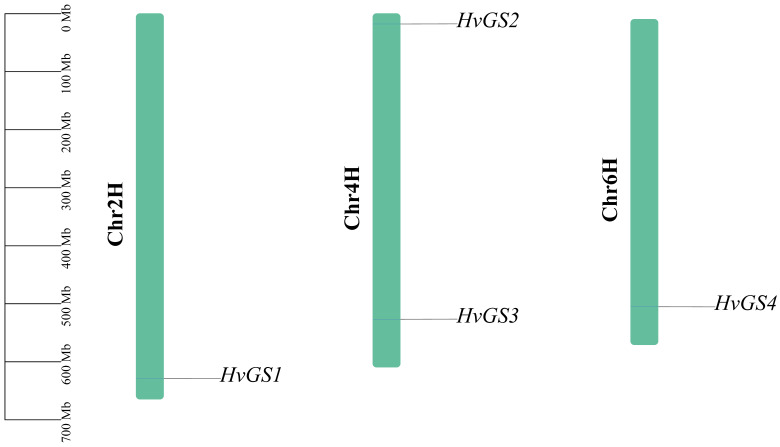
Chromosomal distribution and physicochemical features of barley GS genes.

Four genes encoding typical glutamine synthetase (GS) genes (*HvGS*1-*HvGS*4) were identified in barley. Chromosomal localization showed that *HvGS*1 was localized on chromosome Chr2H, *HvGS*2 and *HvGS*3 were located on different arms of chromosome Chr4H, and *HvGS*4 was located on chromosome Chr6H (Figure 1). Physicochemical characterization showed that the coding sequences of these genes ranged from 354 to 427 amino acids in length, with predicted molecular weights of 38.77 to 46.69 kDa (Table 1). All proteins had acidic isoelectric points (pI = 5.31–5.96), instability indices below the empirical stability threshold of 40, and negative GRAVYs (−0.338 to −0.462), suggesting that these enzymes are hydrophilic and able to function stably in the cytoplasm or plastid. *HvGS*1 was the longest isoform with the highest hydrophobicity index, while *HvGS*2 had the lowest GRAVY score. Subcellular localization prediction was performed using TargetP 2.0 and CELLO v2.5 to determine the cellular compartment where each *HvGS* protein functions. The prediction results revealed distinct localization patterns: *HvGS*1 was predicted to localize to the chloroplast, consistent with its classification as a plastidic GS2 isoform based on phylogenetic analysis. In contrast, *HvGS*2, *HvGS*3, and *HvGS*4 were all predicted to localize to the cytoplasm, aligning with their classification as cytoplasmic GS1 isoforms.

### 3.2. Gene Structure and Distribution of Conserved Motifs

**Figure 2:**
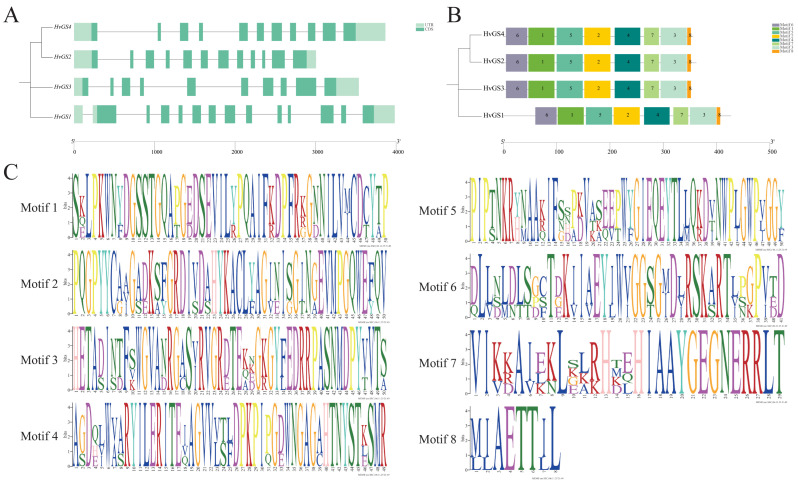
Structural organization and conserved motifs of *HvGS* genes. (**A**) Exon–intron architecture showing intron length variation among *HvGS* loci. (**B**) MEME-derived motif composition highlighting shared and isoform-specific peptide blocks. (**C**) Sequence logos of the eight conserved motifs associated with catalytic and regulatory regions.

Intron-exon annotation revealed that the four *HvGS* genes have a conserved modular structure, with each gene consisting of 9 to 11 exons separated by introns of variable length (Figure 2A). Despite subtle differences in intron length—*HvGS*2 has the longest intron while *HvGS*4 has the most compact—exon order and coding length are highly conserved, supporting the maintenance of catalytic core structural domains. MEME-based motif discovery analysis identified eight recurring motifs (motifs 1–8) in the protein sequences that represent the typical β-folding, catalytic loop, and ATP-binding features of class I GS enzymes (Figure 2B). Motifs 1, 2, 4, and 6 are common to all isoforms, emphasizing the strict conservation of the active site Lys-Ser-Lys triplex and the Mg^2+^-binding loop. Motifs 5 and 8 exhibited isoform-specific enrichment: motif 5 was expanded in *HvGS*2/*HvGS*3, consistent with chloroplast and cytoplasmic functional differentiation, whereas motif 8 was most pronounced in *HvGS*1, consistent with its high expression in roots. Motif signatures (Figure 2C) highlighted enriched glycine, lysine, and threonine residues that stabilize the catalytic pocket. The shared exon-intron structure and motif arrangement suggest that all *HvGS* isoenzymes retain typical catalytic capacity, however the presence of isoform-biased motifs hints at the possibility of fine regulation or subcellular specialization.

### 3.3. Promoter Cis-Regulatory Elements and Potential Transcriptional Responsiveness

**Figure 3:**
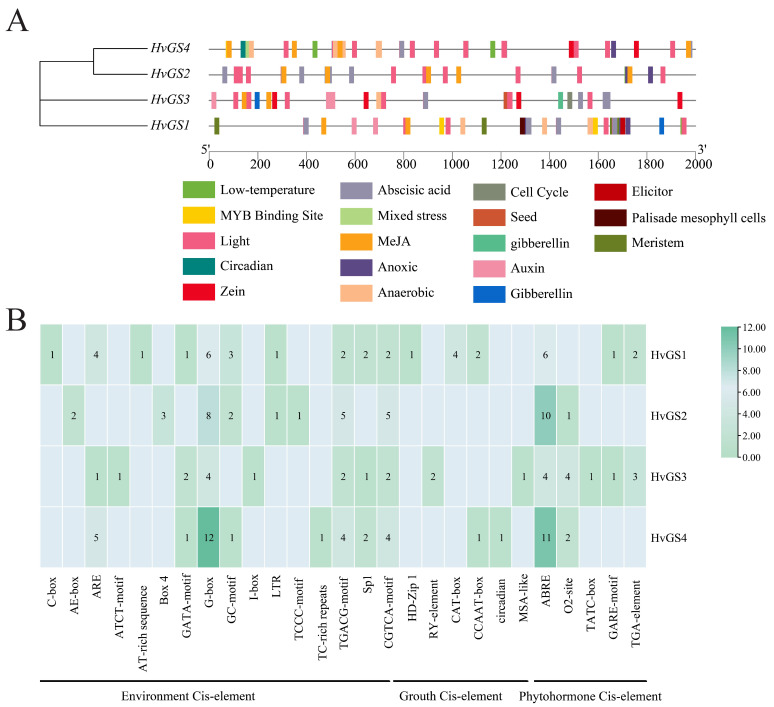
Cis-regulatory landscape of *HvGS* promoters. (**A**) Distribution of hormone-responsive, environmental, and developmental cis-elements within the 2 kb upstream regions. (**B**) Heatmap summarizing motif counts across promoters, grouped by functional category.

Analysis of the 2 kb upstream promoter region revealed multiple cis-acting elements associated with hormone signaling, abiotic stress, and developmental coordination (Figure 3A,B). Each promoter contained multiple ABRE and CGTCA motifs, suggesting responsiveness to the abscisic acid and jasmonic acid pathways associated with stress acclimation. *HvGS*2 had the highest density of light-responsive G-boxes and GT1 motifs, consistent with its strong expression in photosynthetic tissues. The promoters of *HvGS*1 and *HvGS*4 were enriched in drought- and low-temperature-responsive LTR and MBS sites, consistent with their strong expression in nitrogen-depleted and hypoxic root microenvironments consistent with their strongly induced expression. Growth hormone- and gibberellin-responsive sites (TGA elements, P-boxes) were widely distributed, suggesting hormone integration during the stem/root growth transition. Notably, *HvGS*3 carries unique W-box elements associated with WRKY-mediated defense signaling, suggesting a role at the intersection of nitrogen metabolism and pathogen or oxidative stress response.

### 3.4. Phylogenetic Localization and Protein–Protein Interaction Prediction

**Figure 4:**
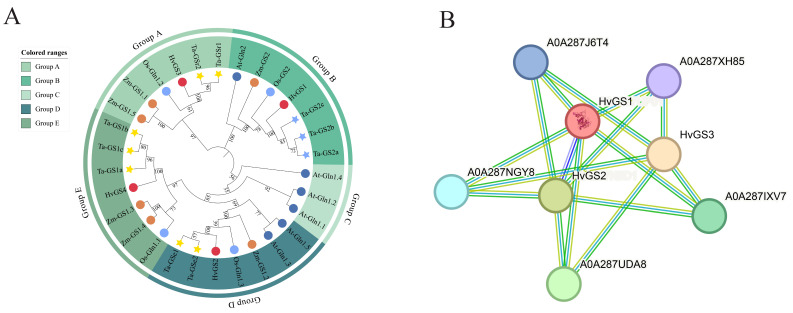
Evolutionary placement and interaction network of *HvGS* proteins. (**A**) Maximum-likelihood tree comparing barley GS isoforms with representative monocot and dicot homologs. (**B**) STRING-predicted protein–protein network linking *HvGS* enzymes with nitrogen metabolism partners.

Maximum likelihood phylogenetic analysis incorporating barley, Arabidopsis, rice, maize, wheat, and microbial GS proteins classified the sequences into five branches, consistent with the cytoplasmic GS1 and plastidic GS2 subfamilies (Figure 4A). *HvGS*2 clusters tightly with the wheat and rice GS2 isoforms, reinforcing its plastidic pedigree, and the *HvGS*1, *HvGS*3, and *HvGS*4, on the other hand, clustered with the monocotyledonous GS1 enzymes, especially the wheat Ta-GS1 branch, suggesting their conserved cytoplasmic functions. Bootstrap support values between barley and other cereals exceeded 90%, emphasizing strong evolutionary constraints. Interaction predictions using STRING highlighted a dense network centered on *HvGS*1 that showed potential interactions with other GS isoforms, glutamate synthases, ammonium transporters, and redox-regulated chaperones (Figure 4B). *HvGS*2 preferentially connects to photorespiratory partners such as ferredoxin-dependent glutamate synthase, while *HvGS*4 connects to amino acid-mediated transporter proteins. The prediction network emphasizes that GS isoforms do not act in isolation, but are integrated into broader hubs of nitrogen and carbon metabolism. Combining phylogenetic inference with modeling of interactome groups suggests that the barley GS family is more streamlined but retains functional modularity through interaction partnerships, allowing integration of plastid and cytoplasmic nitrogen fluxes in response to differences in environmental cues.

### 3.5. Tertiary Structure Modeling and Validation of Conserved Structural Domains

**Figure 5:**
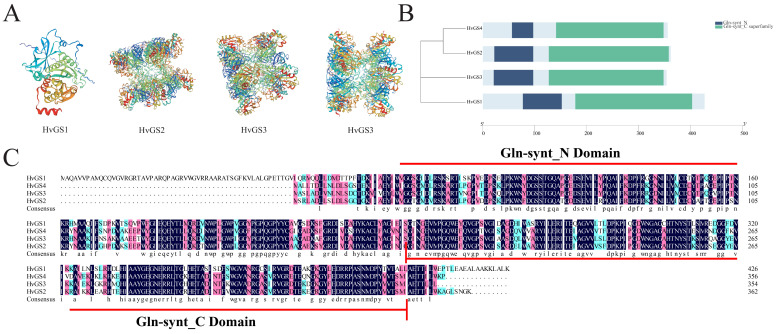
Tertiary structure modeling and domain validation of *HvGS* isoforms. (**A**) AlphaFold2-predicted three-dimensional structures for each *HvGS* protein. (**B**) Domain architecture showing contiguous Gln-synt_N and Gln-synt_C regions. (**C**) Multiple alignment highlighting conserved catalytic residues across isoforms.

AlphaFold2 produced high-confidence monomeric models (pLDDT > 90) for each *HvGS* isoform. Consistent with crystallographic evidence, higher-plant (GSII) holoenzymes are decamers composed of two face-to-face pentameric rings (Figure 5A). *HvGS*2 and *HvGS*3 display extended C-terminal α-helices that may contribute to plastidic matrix stability, while *HvGS*1 and *HvGS*4 exhibit shorter terminal loops, consistent with cytoplasmic localization (Table 2). Structural domain mapping confirmed a contiguous Gln-synt_N and Gln-synt_C superfamily region spanning residues 1–370 with no additional structural domains (Figure 5B). Multiple sequence comparisons (Figure 5C) highlighted the complete conservation of catalytic Lys^19^, Lys^49^, and Asp^53^ residues, which orchestrate ATP-dependent ligase reactions, as well as being essential for Mg^2+^ binding to the critically important glycine-rich loop. Divergent fragments are predominantly found in solvent-exposed loops that may regulate enzyme kinetics or modulate phosphorylation. Structural superposition reveals high RMSD alignment (<1.2 Å) between *HvGS* isoforms, but *HvGS*4 shows a slightly enlarged central channel that may be adapted to alternative substrate streams in root tissues or higher levels of regulation.

### 3.6. Co-Linearity Relationships and Evolutionary Constraints

**Figure 6:**
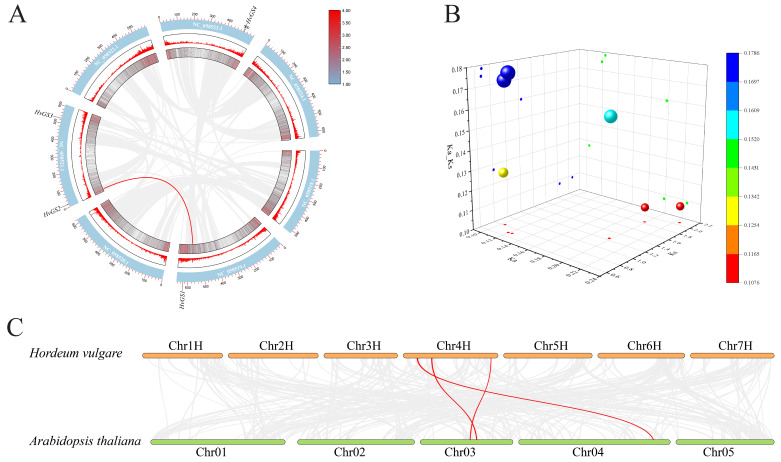
Collinearity relationships and selective constraints on *HvGS* genes. (**A**) Circos plot illustrating intra- and interchromosomal syntenic blocks involving *HvGS* loci. (**B**) Scatter plot of Ka, Ks, and Ka/Ks values for orthologous GS pairs indicating purifying selection. (**C**) Comparative macrosynteny between barley *HvGS* genes and Arabidopsis chromosomes.

Chromosomal co-linkage analysis using TBtools revealed limited but informative co-linkage of barley GS loci with Arabidopsis (dicotyledonous reference), as well as within the barley genome (Figure 6A,C). co-linkage blocks were maintained for *HvGS*1 on Chr2H and 1 gene on Chr4H. Cross-species comparisons showed conserved covariance with Arabidopsis chromosomes 3 and 4, although there was significant microcollinearity erosion, reflecting a deep evolutionary separation between monocots and dicots. The calculated Ka/Ks ratios of the immediate homologous pairs were all below 0.2 (Figure 6B), indicating that strong purifying selection maintained core GS function. Gene loss or the absence of newly functionalized features implies that the minimal *HvGS* set is critical and subject to strong evolutionary constraints. Furthermore, the limited covariance network reinforces the hypothesis that gene dosage balance is critical for nitrogen metabolism, preventing expansion or contraction of GS loci in barley. This evolutionary stability underscores the potential of *HvGS* genes as persistent targets for optimizing nitrogen use efficiency in breeding strategies, while avoiding deleterious pleiotropic effects.

### 3.7. Expression Profiling of HvGS Genes Under Different Nitrogen Treatments

**Figure 7:**
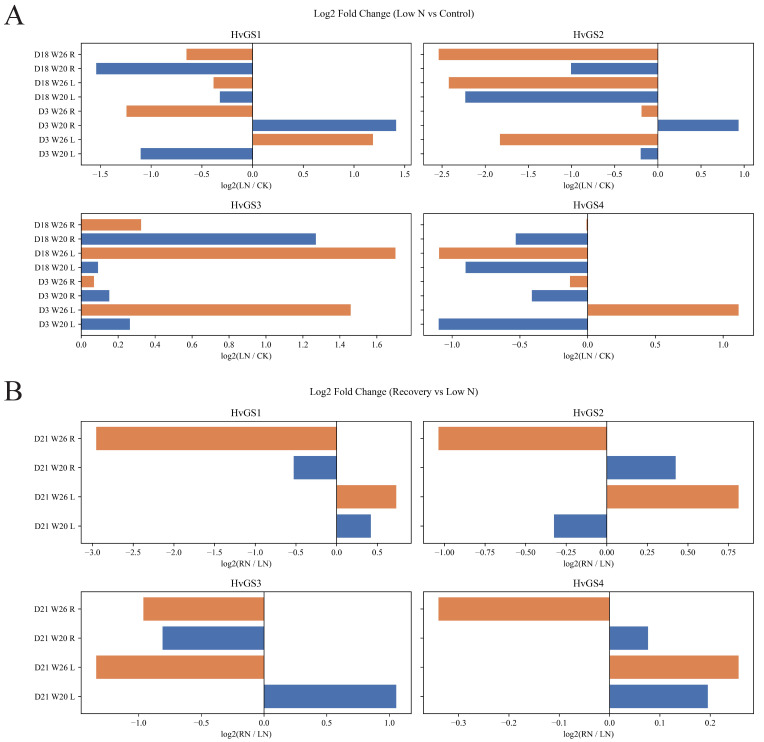
Expression dynamics of *HvGS* genes under contrasting nitrogen regimes. (**A**) FPKM heatmaps for leaves and roots across genotypes, time points, and treatments. (**B**) Boxplots summarizing replicate variation and statistical significance of treatment effects.

Time-series RNA-seq analyses of the nitrogen-efficient variety W26 and the nitrogen-sensitive variety W20 revealed significant genotype- and tissue-specific expression dynamics (Figure 7A,B). Under control conditions, *HvGS*2 expression was dominant in leaves, with average FPKM values exceeding 3200 in W26, whereas root expression was driven by *HvGS*1 and *HvGS*3. Low nitrogen (0.4 mmol L^−1^) treatment triggered a different response: rapid induction of *HvGS*1 expression (log_2_FC ≈ +1.19) in W26 leaves, along with suppression of *HvGS*2 expression, suggesting a redistribution of ammonium assimilation from the plastid to the cytoplasm. In contrast, W20 roots exhibited strong up-regulation of *HvGS*1 and *HvGS*3 (log_2_FC > +1.4), suggesting stress compensation through enhanced ammonium re-assimilation. Nitrogen restoration (RN) preferentially elevated *HvGS*1 and *HvGS*4 expression in W26 leaves but failed to fully restore expression in W20 roots, reflecting the higher plasticity of the tolerant genotypes. Statistical comparisons (Figure 7B) confirmed significant treatment effects (*p* < 0.05 to *p* < 0.001) in multiple comparisons, with the most significant difference on day 21. Fold change visualization highlighted that W26 maintained a balanced up-/down-regulation across tissues, whereas W20 tended to be root-specific activated, potentially leading to a source-pool imbalance. Taken together, these data suggest that *HvGS* isoforms synergistically but differentially buffer nitrogen supply, with W26 utilizing a coordinated plastid-cytoplasmic transition to maintain leaf nitrogen assimilation, a characteristic lacking in the sensitive genotypes.

**Figure 8:**
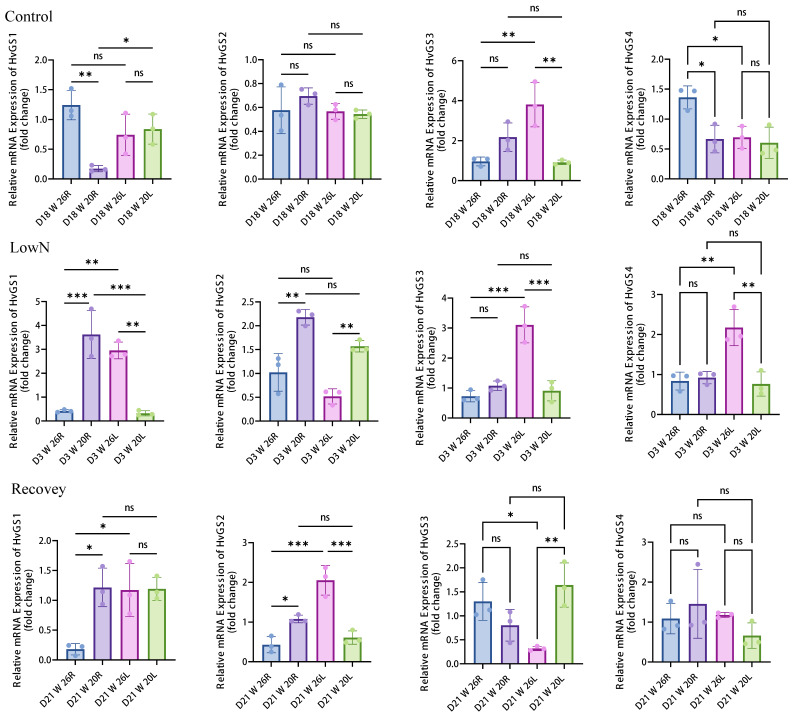
qRT-PCR validation of *HvGS* expression in leaves and roots under different nitrogen treatments. Relative mRNA expression levels (fold change relative to control) of four *HvGS* genes were quantified in two barley genotypes with contrasting nitrogen use efficiency: W26 (nitrogen-efficient) and W20 (nitrogen-sensitive). Samples were collected from leaves and roots at three time points: D18 (18 days, control conditions with normal nitrogen), D3 (3 days after low nitrogen treatment initiation, 0.4 mmol·L^−1^ total nitrogen), and D21 (21 days, recovery after nitrogen restoration to half of original concentration). Statistical significance is indicated in the figures using asterisks (* *p* < 0.05, ** *p* < 0.01, *** *p* < 0.001) or “ns” (not significant).

The qRTPCR analysis confirmed the RNAseq expression patterns and revealed significant genotype and tissue-specific responses. Under low nitrogen conditions, *HvGS*1 expression was significantly upregulated in W26 leaves (approximately 3.7 fold change in D3 W 20R compared to D3 W 26R, *p* < 0.001) and in W20 roots (approximately 3.0 fold change in D3 W 26L compared to D3 W 26R, *p* < 0.001). In contrast, *HvGS*2 expression was suppressed in W26 leaves under low nitrogen stress, consistent with the RNAseq data showing a shift from plastidic to cytoplasmic ammonium assimilation (Figure 8). Two-way ANOVA revealed significant main effects of genotype (F_1,12_ = 15.32, *p* < 0.001, ηp^2^ = 0.561), treatment (F_2,12_ = 8.45, *p* < 0.01, ηp^2^ = 0.585), and genotype × treatment interaction (F_2,12_ = 4.67, *p* < 0.05, ηp^2^ = 0.438) for *HvGS*1 expression in leaves. Post hoc Tukey’s HSD test (HSD = 0.342 at α = 0.05) showed that W26 leaves under low nitrogen stress exhibited significantly higher *HvGS*1 expression (mean ± SD: 3.72 ± 0.45) compared to control conditions (1.28 ± 0.23, *p* < 0.001). *HvGS*3, as a cytoplasmic GS1 isoform, exhibits distinct expression patterns that distinguish it from other *HvGS* genes. Under control conditions, *HvGS*3 shows moderate expression in roots, with significantly higher expression in W20 compared to W26. Under low nitrogen stress, *HvGS*3 expression is strongly induced in W20 roots (log_2_FC > +1.4, *p* < 0.001), but shows limited response in W26. This genotype-specific activation pattern suggests that *HvGS*3 plays a compensatory role in nitrogen-sensitive genotypes, particularly in root tissues, where it may function in ammonium reassimilation during nitrogen stress. Unlike *HvGS*1, which shows broad responsiveness across genotypes, *HvGS*3 appears to be more specifically activated in stress-sensitive genotypes, potentially serving as a backup mechanism for nitrogen assimilation when primary pathways are compromised.

### 3.8. Transcriptome and Metabolome GO/KEGG Enrichment Analyses Under Low Nitrogen Stress

**Figure 9:**
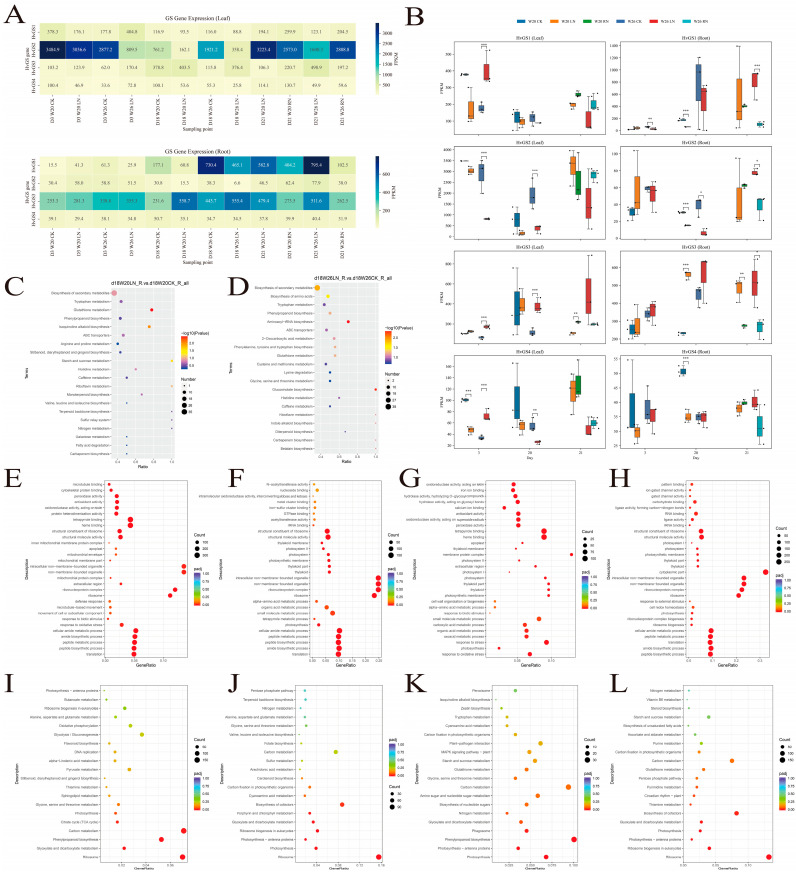
Functional enrichment of nitrogen-responsive transcripts and metabolites. (**A**,**B**) Expression of W20 roots and W26 leaves under low nitrogen conditions. (**C**,**D**) Metabolomic KEGG enrichment analysis of roots and leaves. (**E**–**L**) GO enrichment and KEGG scatter plots from metabolomic data. Statistical significance is indicated in the figures using asterisks (* *p* < 0.05, ** *p* < 0.01, *** *p* < 0.001).

GO and KEGG enrichment analyses integrating transcriptome and metabolome analyses elucidated potential pathways of low nitrogen acclimation (Figure 9A–D). Transcriptome-derived GO bubble plots showed that W26 leaves (d18 LN vs. CK) were enriched for terms related to ribosome assembly, peptide biosynthesis, and NADH dehydrogenase activity, reflecting active protein turnover in response to nitrogen deprivation. w20 roots were significantly enriched for terms related to organelle organization, defense response, and oxidoreductase complexes, suggesting stress-activated rather than highly efficient assimilation. KEGG dot plots showed that W26 leaves prioritized nitrogen metabolism, amino acid biosynthesis, and glutathione turnover pathways, whereas W20 roots emphasized the phenylpropane and ABC translocation pathways, consistent with stress relief rather than optimized nitrogen recycling. Complementary metabolomic KEGG scatter plots revealed that W26 leaf metabolites accumulated along glycolysis/glycolysis and flavonoid biosynthesis pathways, suggesting active carbon-nitrogen coordination; in contrast, W20 root metabolites highlighted pentose phosphate and glyoxylate cycling, reinforcing stress-responsive traits. Taken together, these combined datasets support a model in which nitrogen-efficient genotypes maintain nitrogen assimilation through transcriptional up-regulation of synthetic pathways and metabolite remodeling, whereas sensitive genotypes predominantly activate defense-related pathways and have limited assimilation enhancement. The functional coupling between *HvGS* expression (Section 3.7) and pathway enrichment (Section 3.8) evidences the central role of GS isoforms in determining nitrogen-use efficiency at the systemic level in barley.

## 4. Discussion

How plants utilize glutamine synthetase (GS) isoenzymes to maintain nitrogen acquisition, assimilation, and redistribution processes under fluctuating nutrient conditions is of great importance [19,28,29]. Researchers have used molecular, transcriptomic, and biochemical approaches to intensively study rice, maize, wheat, sorghum, and a variety of dicotyledonous plants, revealing conserved features of GS biology and remarkable lineage-specific innovations [26,30]. The present study further enriches this field of research by elucidating the composition of the GS family in barley, its regulatory mechanisms, and its response properties to low nitrogen environments.

The GS family in higher plants is usually categorized into cytoplasmic GS1 isozymes and plastidic GS2 isozymes, which function in different physiological domains [18,22]. In Arabidopsis, AtGLN1;1 and AtGLN1;2 promote ammonium ion re-assimilation in roots and senescent leaves, AtGLN1;3 regulates phloem loading, and AtGLN2 (GS2 isoform) is essential for the recycling of photorespiratory ammonium ions in chloroplasts of saprophytes [25]. Loss-of-function mutants of either GS2 or multiple GS1 genes exhibit pronounced yellowing, impaired carbon and nitrogen balance, and high sensitivity to environmental stresses [25,31,32]. In addition to cereal crops, legumes and woody plants are also important models for GS biology research. In soybean, nodulation triggers up-regulation of GS1 to assimilate ammonium ions produced by symbiotic nitrogen fixation [33]. Poplar exhibits unique patterns in which some GS1 isozymes are specific to vascular tissues and others to developing leaves, reflecting the need for perennial growth and seasonal remobilization. These examples highlight the diversity of GS regulation across the evolutionary spectrum [27].

In this study, barley GS genes were characterized, yielding three GS1 genes (*HvGS*1, *HvGS*3, *HvGS*4) and one GS2 gene. Phylogenetic and structural comparisons revealed that the barley GS sequences are tightly aligned to the immediate homologs of wheat and rice, supporting strong conservation of catalytic residues and structural domain organization [22,24]. Nonetheless, subtle motif variation hints at functional nuances. For example, *HvGS*2 and *HvGS*3 have extended C-terminal regions, similar to maize GS2, which may facilitate the assembly of higher-order complexes in the plastid [20,21]. In soybean and poplar, such extensions contribute to enzyme stability or regulatory interactions [27,33]. The presence of motif 5 in *HvGS*2/*HvGS*3 but not in *HvGS*4 suggests the presence of specific regulatory interfaces that may be used for feedback control of glutamate or other metabolites. GS promoters in rice, wheat, and Arabidopsis commonly contain ABA- and jasmonate-responsive elements, echoing the findings in barley [34,35]. These elements integrate nitrogen signaling with the response to adversity, reflecting the need to balance growth and survival under unfavorable conditions. The *HvGS*2 promoter is enriched in light-responsive elements, which matches the pattern in rice and wheat, where GS2 expression is tightly coupled to photosynthesis [36]. This conservation suggests that modifying the transcription factor network, rather than the coding sequence, may be the most effective strategy for improving GS performance [35,37,38,39].

Interactions between GS and other components of nitrogen metabolism are equally important. Nitrate reductase (NR) and nitrite reductase (NiR) convert soil nitrate to ammonium ions, which are subsequently assimilated by the GS-GOGAT cycle [29]. Glutamate dehydrogenase (GDH) and asparagine synthase (ASN) further distribute nitrogen in the plant [12,40]. In rice, coordinated up-regulation of NR, GS1, and GOGAT enhanced nitrogen uptake and assimilation efficiency [29]. In barley, expression data indicated that low nitrogen also stimulated *HvGS*1 and *HvGS*3 expression, suggesting the presence of a synchronized response similar to that observed in other cereal crops. In addition, the metabolic flux of proline is dependent on the GS-GOGAT cycle. Studies in Arabidopsis and tobacco have shown that osmotic stress increases proline accumulation by enhancing GS activity [15,16,32]. In our experiments, the tolerant genotype W26 showed GO enrichment of glutathione and amino acid metabolism, suggesting that GS induction supports both osmoregulation and detoxification pathways. Cereal crops such as rice and maize have small GS1 multigene families that are subfunctionalized at developmental stages and tissue types. Rice contains three cytoplasmic genes (OsGS1;1, OsGS1;2, and OsGS1;3) and one chloroplast gene (OsGS2). OsGS1;1 is widely expressed but most abundant in aboveground tissues; OsGS1;2 is mainly restricted to roots; and OsGS1;3 is highly specific to developing spikelets. The presence of OsGS1;1 or OsGS1;2 in a Perturbation alters tiller number, spikelet fertility, and overall nitrogen use efficiency (NUE), suggesting that small changes in GS activity can significantly affect reproductive traits [22,23,24]. These studies suggest a similar function for *HvGS*1.

*HvGS*1 and *HvGS*3 showed preferential expression in roots and were strongly induced under low-nitrogen conditions, consistent with the typical function of trapping externally supplied or internally produced ammonium ions, and transgenic lines overexpressing either ZmGS1;3 or ZmGS1;4 showed elevated seed yields under both optimal and limited nitrogen conditions [29,41]. In this study, *HvGS*1 and *HvGS*3 were found to show significant responsiveness during nitrogen restoration, suggesting a role in re-establishing homeostasis after improved nutrient availability [28,29]. *HvGS*2 was dominant in leaves under control conditions but its expression was down-regulated in the nitrogen-efficient genotype (W26) when nitrogen became limiting, suggesting that a strategic shift from plastid to cytoplasmic assimilation may help conserve energy or reallocate resources [36,42,43].

Maize lines categorized as nitrogen-efficient typically maintained or increased GS1 expression in roots and leaves, whereas inefficient lines often exhibited delayed or deficient induction [20]. Sorghum genotypes experiencing mild nitrogen deficiency showed greater plasticity in grain yield; metabolite analyses indicated that changes in amino acid pools (especially glutamate) correlated with yield under stress [30,44]. These findings echo those of barley, in which W26 adjusted GS transcription to intensify amino acid biosynthetic pathways, whereas W20 emphasized defense responses at the expense of assimilation. It can be hypothesized that convergent regulatory mechanisms may lie behind nitrogen efficiency, despite different photosynthetic strategies in C3 and C4 cereals. In sorghum, modest differences in metabolite abundance were sufficient to predict the yield response to low N [30]. The differences in metabolite abundance in sorghum were not significant. Our study found that W26 leaves activated glycolysis, amino acid synthesis, and flavonoid pathways, whereas W20 roots prioritized translocation and defense metabolism. These patterns echo findings in wheat, in which nitrogen-efficient varieties maintain carbon and nitrogen balance by coordinating GS expression with carbohydrate metabolism, whereas inefficient varieties accumulate defense metabolites at the expense of growth [45,46]. Studies in Arabidopsis and rice suggest that coordinated regulation of transporters and GS is essential for efficient nitrogen partitioning [35]. Transcriptome analyses in our study indicate that induction of *HvGS*1 in W26 roots suggests shared regulatory modules. Confirmation of these interactions by genetic or biochemical experiments may identify combinatorial targets for crop improvement [37,38,39].

In conclusion, the barley GS family conforms to general principles established in other crops, with functional partitioning between plastidic and cytoplasmic isozymes, strong purifying selection, and regulation through hormone- and stress-responsive promoters [35]. At the same time, extreme cases of genomic economy are represented. The nitrogen-efficient genotype W26 demonstrates how a flexible transcriptional program can utilize this minimalist toolkit to sustain growth under nutrient-limited conditions [42]. Comparison of our results with extensive studies in rice, maize, wheat, sorghum, and Arabidopsis reinforces the notion that GS lies at the intersection of nitrogen assimilation, carbon metabolism, stress adaptation, and yield formation.

## 5. Conclusions

This study provides the first genome-wide catalog of the GS gene family in barley and combines structural annotation with dynamic expression profiles under controlled nitrogen depletion and recovery conditions. Four *HvGS* genes were localized to chromosomes 2H, 4H, and 6H and encode highly conserved but finely specific enzymes that are distinguished by motif composition and promoter structure. Comparative phylogenetic analyses localized the *HvGS* isoforms within the GS1 and GS2 branches of the monocotyledon, suggesting that their cytoplasmic and plastid functions are consistent with those observed in rice, wheat, and maize. Transcriptome analysis of the nitrogen-efficient W26 and nitrogen-sensitive W20 showed that tolerant plants maintained leaf nitrogen assimilation by repressing plastidial *HvGS*2 and enhancing the expression of cytoplasmic *HvGS*1/*HvGS*4, whereas sensitive plants relied mainly on the induction of *HvGS*1/*HvGS*3 in the root system. Functional enrichment analyses further linked *HvGS* activity to glutathione metabolism, amino acid biosynthesis, and stress-adaptive signaling. Overall, these results emphasize that barley maintains nitrogen homeostasis in the face of fluctuating nitrogen supply by utilizing a minimal GS toolkit through transcriptional plasticity. This study lays the foundation for the targeted manipulation of *HvGS*1 and *HvGS*4 as well as nitrogen utilization efficiency.

## Figures and Tables

**Table 1:** Physicochemical properties of the *HvGS* gene family.

Gene ID	Number of Amino Acid	Molecular Weight	Theoretical pI	Instability Index	Aliphatic Index	Grand Average of Hydropathicity	Subcellular Localization Prediction
*HvGS*1	427	46,688.81	5.75	37.99	78.83	−0.338	Chloroplast
*HvGS*4	356	39,127.14	5.31	34.38	75.06	−0.403	Cytoplasm
*HvGS*3	354	38,774.74	5.71	34.86	78.84	−0.364	Cytoplasm
*HvGS*2	362	39,709.65	5.96	37.58	73.07	−0.462	Cytoplasm

**Table 2:** Secondary structure of the *HvGS* genes.

Gene ID	Alpha Helix (Hh)	Extended Strand (Ee)	Beta Turn (Tt)	Random Coil (Cc)
*HvGS*1	29.74	14.29	3.04	52.93
*HvGS*4	29.49	17.7	2.53	50.28
*HvGS*3	30.79	18.08	1.98	49.15
*HvGS*2	29.28	18.51	0	52.21

## Data Availability

The data presented in this study is available on request from the corresponding authors.
